# Identifying Sexual and Labor Exploitation among Sheltered Youth Experiencing Homelessness: A Comparison of Screening Methods

**DOI:** 10.3390/ijerph16030363

**Published:** 2019-01-28

**Authors:** Salina Mostajabian, Diane Santa Maria, Constance Wiemann, Elizabeth Newlin, Claire Bocchini

**Affiliations:** 1 Baylor College of Medicine, Houston, TX 77030, USA; smostaja@gmail.com (S.M.); cmwieman@texaschildrens.org (C.W.); cebocchi@texaschildrens.org (C.B.); 2 Texas Children’s Hospital, Houston, TX 77030, USA; 3 Cizik School of Nursing, University of Texas Health Science Center at Houston, Houston, TX 77030, USA; 4 McGovern Medical School, University of Texas Health Science Center at Houston, Houston, TX 77030, USA; Elizabeth.W.Newlin@uth.tmc.edu

**Keywords:** Human trafficking, youth homelessness, sexual exploitation, labor exploitation, screening tool

## Abstract

Human trafficking is a significant and growing public health concern. Subgroups of adolescents and young adults are particularly vulnerable to human trafficking, especially youth who are unstably housed or homeless. While youth experiencing trafficking come into contact with the healthcare system, they are often not identified during routine assessment due to lack of specific inquiry and low disclosure. Therefore, we utilized a mixed-methods study design to assess the differences in the identification of human trafficking among youth experiencing homelessness (*n* = 129) between a standard psychosocial assessment tool and a human trafficking specific assessment tool. Findings indicate that the tool developed to specifically assess for human trafficking was more likely to identify youth experiencing sexual and labor exploitation, as well as the risk factors for human trafficking. Secondly, youth reported that mistrust of the system, fear of involving the police if reported, not wanting to interact with the mental healthcare system, and stigma are barriers to disclosing human trafficking. In conclusion, healthcare providers caring for youth experiencing homelessness should adopt improved screening tools for human trafficking to reduce the risk of missed opportunities for prevention and treatment among this high-risk population of youth.

## 1. Background

Human trafficking is a significant and growing public health concern among adolescents and young adults. It is defined as the recruitment, transportation, transfer, harboring or receipt of persons, using force or threat of coercion, abduction, fraud, deception or payments, or benefits to achieve control over another person for exploitation (The United Nations Office on Drugs and Crime). Sex trafficking is defined as a commercial sex act that involves force, fraud, or coercion, or the inducement of a minor to perform sexual acts. In a study of 810 youth from 16 community sites across the U.S., the overall prevalence of child sex trafficking was 11.1% with rates ranging from 6.3% among child advocacy center patients, 13.2% among emergency department patients, and 16.4% among youth presenting at a teen clinic [1]. The Trafficking Victims Protection Act of 2000 defines labor trafficking as the recruitment, harboring, transportation, provision, or obtaining of a person for labor or services, through the use of force, fraud, or coercion for the purpose of subjection to involuntary servitude, peonage, debt bondage, or slavery. Labor trafficking involves recruiting, harboring, moving or obtaining a person by force, fraud, or coercion for the purposes of involuntary servitude, debt, bondage, or sexual exploitation. 

Texas has the second highest number of cases of human trafficking reported right after California (The United Nations Office on Drugs and Crime). In a prevalence study across the state of Texas in 2016, there were an estimated 313,000 people experiencing human trafficking, including 79,000 minors and youth experiencing sex trafficking. It is estimated that sex trafficking costs the state of Texas $6.6 billion and that traffickers exploit $600 million from victims of labor trafficking [2]. 

Subgroups of adolescents and young adults are particularly vulnerable to human trafficking, especially sex trafficking, including youth involved in the foster care or juvenile justice systems, those experiencing abuse, youth who run away, and those who are unstably housed or homeless [2,3,4,5]. A New York City-based study found that 75% of sex trafficking survivors had prior child welfare or foster care involvement [4]. In a retrospective study of patients aged 12–18 years presenting at a pediatric emergency department or child protection clinic, youth who were victims of commercial sexual exploitation were more likely to report running away from home, involvement with child protective services, and interaction with law enforcement [5]. 

Youth experiencing homelessness are at particularly high risk for human trafficking. The Administration for Children and Families at the U.S. Department of Health and Human Services issued new guidance on child trafficking to child welfare systems and runaway and homeless youth programs because of increased vulnerability to trafficking for youth who have experienced prior abuse or who have run away from home. In a study conducted in 10 homeless youth shelters across the U.S. among youth aged 17 to 25, 19% reported experiencing human trafficking [6]. Of those trafficked, 76% reported sex trafficking, 43% had been trafficked for labor, and 18% had experienced both sex and labor trafficking [6].

Youth victims of trafficking often come into contact with various service systems and institutions, such as the police, judges, therapists, teachers, and the healthcare system, but face significant barriers to being identified as victims and to accessing services that could help them find safety and treatment. Healthcare providers that work with adolescents and young adults have an important role in identifying, responding to, and advocating for youth experiencing human trafficking [7]. Evidence suggests that victims of human trafficking interact with the healthcare and social services systems where they can be identified. A study among adolescents found that 43% of trafficking victims had visited a healthcare provider in the last two months [5]. In a small retrospective study, 81% of children who were victims of domestic sex trafficking had seen a healthcare provider within the year before being identified and referred for services [8]. 

Tools that show promise for identifying human trafficking across various locations have been developed, though the specificity and sensitivity vary across sites where youth present. West Coast Children’s Clinic developed the Commercial Sexual Exploitation-Identification Tool (CSE-IT) and determined the validity of this measure during a 15-month period of pilot testing in juvenile justice, community-based organizations, and child welfare organizations [9]. Criterion validity was determined by comparing data collected using the CSE-IT and the validated Crisis Assessment Tool/Childhood Severity of Psychiatric Illness [9]. The psychometric properties, including factor structure and reliability of the CSE-IT, were further determined using Exploratory Factor Analysis [9]. The CSE-IT is disseminated in a train-the-trainer model to promote fidelity. It has been introduced in five states and as of January 2018 it has been used to screen over twenty thousand youth [9]. The Trafficking Victims Identification Tool (TVIT), developed by the Vera Institute of Justice, was validated for use in adults and youth to detect either sex or labor trafficking in community-based settings [10]. The TVIT is unique in that it has been translated and validated for use in several languages [10]. The Urban Institute designed the Human Trafficking Screening Tool (HTST) to be used with young people involved in the child welfare and runaway and homeless youth systems [11].

Several screening tools exist for use in healthcare specific settings, such as a teen clinic or emergency department [1,12,13,14]. Only recently has an instrument been developed and tested for use with the youth population across multiple health care settings and sites [1]. A short and easy to administer screener developed to identify child sex trafficking victims in healthcare settings found that the youth who presented to the emergency departments had the highest likelihood of being identified (84.4%), followed by youth presenting at child advocacy centers (57.5%), and patients from a teen clinic (2.0%) [1]. It should be noted that no healthcare setting specific screening tool has been evaluated with non-English speaking youth and additional research is needed to validate existing measures. Further, while the HTST has been developed specifically for youth experiencing homelessness, no studies have been done to compare it to usual care assessment tools often used in these settings. 

There are several challenges with the identification of youth experiencing human trafficking among those seeking services at a homeless youth shelter. Youth experiencing homelessness face significant social stigma and often have difficulty accessing health and social services [15,16]. Therefore, healthcare providers caring for homeless youth need to be able to accurately identify victims of trafficking to link them to needed services. Typical assessment methods include interview style questioning and use of a psychosocial assessment tool [17]. However, little is known about whether these assessment tools are effective at screening for the risk factors for human trafficking or sensitive to either sexual or labor exploitation. 

### Aim

The primary aim of this study was to compare the prevalence of sexual and labor exploitation and trafficking related risks and experiences identified using a structured survey versus a routine psychosocial assessment performed by physicians at a shelter for youth experiencing homelessness. Secondary aims of this study were to identify gaps in knowledge about human trafficking and available resources, as well as barriers to disclosing human trafficking and seeking help. These aims were accomplished using a mixed methods approach involving surveys, medical record reviews, and focus group discussions. 

## 2. Methods

This study is part of a larger 3-site study to pilot test a structured human trafficking screening tool (HTST) aimed at identifying trafficking experiences in youth involved in the child welfare system or receiving runaway/homeless youth services. In this paper, we present findings from the largest shelter for homeless young adults in Houston, Texas.

A quantitative dominant mixed-method study design was used, including a self-administered survey or a provider-administered survey, a retrospective review of medical records to abstract data from the routine psychosocial assessment performed by physicians, and focus group discussions [18]. The study was conducted in accordance with the Declaration of Helsinki, and the protocol was approved by the Institutional Review Board for Human Subjects Research at Baylor College of Medicine and University of Texas Health Science Center (H-38073, H-38836), as well as the Urban Institute (08800-052-00). Given the vulnerabilities of youth experiencing homelessness and the sensitive nature of the questions, all survey data were collected anonymously and informed consent for the survey portion was obtained verbally for added privacy protection. All focus group participants provided written informed consent.

### 2.1. Participant Recruitment

Participants were recruited from a single shelter providing short and long-term housing and services to youth experiencing homelessness ages 18–21 years. From May to November 2016, each youth evaluated at the shelter clinic was invited to participate in a survey. Eligible participants spoke English or Spanish, passed a cognitive screener, and provided verbal consent. The screener was administered by a healthcare provider and included assessment of orientation to time and place, as well as short term memory. Exclusion criteria were: failure to pass the cognitive screening test, or being unable or unwilling to give consent. From May through November 2016, the medical record of every youth evaluated at the shelter clinic was reviewed to extract data that could indicate possible trafficking. 

### 2.2. HTST Administration

The HTST was available in English and Spanish via Qualtrics software with audio computer-assisted self-interview capability. Participants were randomly assigned via 3:1 ratio (self-administered survey: provider-administered survey) based on the participant’s month of birth being in the first three quarters (self-administered arm) or the last quarter of the year (provider administered arm). Each participant completing the self-administered survey was provided complete privacy in a closed-door clinic exam room. All provider-administered surveys were administered by the primary researcher who was trained in the administration of the HTST. Participants completed the survey or interview within 20–30 minutes. Results comparing the mode of administration of the HTST were reported as part of the parent study [11]; no differences by mode of administration were found. Thus, for the purpose of data analysis for our study, the results of self-administered and provider-administered surveys were combined to compare to the medical record review of the routine psychosocial assessment. 

Participants received a $25 gift card for completing the HTST and were assessed for mental health safety by asking about any current negative feelings, thoughts of self-harm, suicidal ideation, or other emotional distress experienced in general or as a result of their participation. Access to mental health providers who assessed youth per clinic protocols was made available in two cases where youth reported emotional distress regarding their past experiences. All participants were provided with written information about human trafficking, local resources, as well as the National Human Trafficking Hotline. 

### 2.3. Structured Screening Tool

The HTST was developed by the Urban Institute to assess for human trafficking experiences among youth involved in the child welfare system or receiving runaway/homeless youth services [11]. Survey measures were divided into the following categories. Socio-demographic items assessed race, gender, sexual orientation, education level, and truancy in the past year. Issues related to birthplace and immigration were assessed, including immigration process, forced immigration, outside help, payments and abuse/threats. Items also asked about family relationships, shelter use, history of running away, and the onset of homelessness. To assess for labor exploitation, work was defined broadly as any service rendered voluntarily or involuntarily in exchange for something of value from an employer, friend, family member or stranger. Questions included types of work with specific questions focusing on trading sex with/without third party involvement. Financial, emotional, physical, or sexual abuse, and threats at a work place or by superiors were assessed. The survey asked youth about other life experiences, including a history of arrest, past month history of prescription and illicit drugs, and binge drinking. Finally, help seeking was assessed using two items that asked about attempts to get help and sources of help, including family members, friends, health agencies, and social institutions. 

### 2.4. Retrospective Medical Records Review

Baseline data for detection rates of potential trafficking experiences were collected by reviewing the medical records of all residents evaluated at the clinic during the study period. Data abstraction was focused on information documented in the medical record by primary care, adolescent medicine physicians, fellows, or attendings, and psychiatrists, including diagnoses and the HEEADSSS assessment; as well as notes recorded by nurses during initial clinic intake. The HEEADSSS assessment is a psychosocial review of systems which covers Home environment, Education and employment, Eating, peer-related Activities, Drugs, Sexuality, Suicide/depression, and Safety from injury and violence” [19]. Most questions were asked in an open-ended format and included demographic data, education level, home environment, history of child abuse and previous foster care sytems involvement, sexual practices, mental health, substance abuse, and safety. Medical records did not include specific questions regarding force, fraud, or coercion in work setting or those involving trade sex; thus, the details and documentation of those items varied across the records. 

### 2.5. Focus Group Discussions

Focus group discussions were conducted at the shelter from August 2016 to March 2017. Participants were recruited by researchers through announcements at regularly scheduled group meetings held at the shelter attended by most residents. Each participant was allowed to participate once and was compensated with a $10 gift card as well a resource sheet on human trafficking. The focus group discussions were designed to assess knowledge of youth regarding definitions of human trafficking, needs of survivors, perceived barriers to seeking services, and knowledge regarding available services. Specifically, the semi-structured guide asked (1) What youth had heard or know about human trafficking, (2) When and how did they first hear about human trafficking, (3) Thoughts on the definitions of trafficking, (4) Who youth knew who had encountered any similar situations, (5) How someone may become a victim of trafficking, (6) What resources would someone who has gone through these experiences need, (7) What resources they know of, and (8) What would prevent someone experiencing trafficking from going to the police, community centers, or a clinic for help.

### 2.6. Data Analysis

**Quantitative analysis.** Descriptive statistics of data collected from surveys and via medical record review were used to describe the study sample and to generate prevalence estimates of events associated with higher potential for experiencing trafficking. The primary endpoint of trafficking risk factors or experiences detected by medical record review vs. the HTST were compared using a test of two proportions (STATA 13, Stata Statistical Software, Release 13, StataCorp LP., College Station, TX, USA), as the HTST were completed anonymously and therefore not linked to data collected from individual medical records.

**Qualitative analysis.** For the qualitative portion, analysis was conducted using descriptive and thematic content analysis [20]. First, transcriptions from the focus group discussions were reviewed to develop a codebook. Next, two researchers identified themes independently and reviewed for comparison to determine final themes. Finally, the researchers identified relevant exemplar quotes that matched with the themes. The data were analyzed and organized using ATLAS.ti software (ATLAS.ti Scientific Software Development GmbH, Berlin, Germany). The qualitative data was analyzed specifically to identify gaps in knowledge of youth about human trafficking, potential available resources, and disclosure barriers to inform the development of effective primary and secondary prevention interventions. Finally, triangulation of the qualitative and quantitative data was used to further describe the data [18].

## 3. Results

### 3.1. Sample Description

Of the 129 shelter residents evaluated at the clinic during the study period, 121 (93.8%) gave verbal consent to participate in the screening study. Of these, one was excluded based on low cognitive function as assessed by a score <4 on the cognitive screener. The medical records of all 129 patients were reviewed. Demographic characteristics of the sample that completed the survey and the sample for whom medical records were reviewed are presented in Table 1. There were no statistically significant differences in the characteristics of the sample between the methods of screening. The sample averaged 19 years of age (range 18–21 years), 45% female, 75% African-American or mixed-race, and majority were born in the United States and raised in Texas, with 11 (9%) born outside the U.S. One participant reported being transgender on either the survey or medical record review, and about 75% reported being heterosexual. About half reported having completed 12th grade or being in higher education, and 25% reported being currently pregnant or parenting. The sample composition approximates the population of youth served by the shelter. Covenant House Texas provided shelter to 463 individual youth in 2017 of whom 46% were female, 54% male, 68% African American, 18% Caucasian, 13% Hispanic, and 1% Asian [21].

Data on mental health disorders and sexual risk behaviors were available in the medical records only. Sixty-nine (53.5%) participants had a psychiatric diagnosis recorded, with 43 (59.0%) having more than one diagnosis; of which 34 (49.3%) reported as depression. Forty-three (59.4%) had a history of psychiatric hospitalization, and 32 (42%) had at least one prior suicide attempt. Twenty-five participants (19.4%) reported 10 more sexual partners (mean = 6.4 ± 10) in their lifetime, 37 (28.7%) had a prior history of a sexually transmitted infection and three (2.3%) had positive HIV status. Only 20 (15.5%) had ever used hormonal contraception while 82 (63.6%) reported condom use. 

### 3.2. Housing and Justice-System Involvement

Higher rates of involvement in the justice system (*p* < 0.01) and foster care system (*p* = 0.02), as well as running away from home and being kicked out of home by the family (*p* < 0.01) were reported via the HTST compared to the medical record review (Table 1). Forty-four percent of participants reported ever being arrested on HTST compared to 13% in the medical records. Of those who reported arrest on HTST, 39% were arrested as a child, 41% arrested as an adult, and 30% reported more than three arrests. Thirty-five percent of participants reported a history of foster care via HTST compared to 22.5% in the medical records, with an average age at placement of seven-eight years old. Based on HTST data, 12.5% reported greater than three foster care placements and 5% reported having spent greater than five years in foster care. Seventy-one percent reported being kicked out of their home by their family on HTST versus 48% in the medical records, with the average age ranging from 16 to 18 and the youngest age being 13 years. Twenty-nine percent reported being kicked out by family three or more times. Similarly, 45% reported ever running away from home on HTST compared to 7% on the medical records, with an average age of 14, and the youngest at age 11 years. Eighteen percent reported running away from home three or more times. Forty-two percent of participants reported both having ran away from home and being kicked out by family in the past. 

In the HTST, participants were asked about where they slept most nights in the previous month and their perceived safety; however, this information was not specifically asked during the psychosocial assessment and therefore was not recorded in the medical record. About half of the participants were literally homeless and staying on the streets. Fifty percent reported staying with family, friends, couch surfing, staying at a foster or group home, or in a rental unit or hotel. Twenty-nine percent reported having been in treatment facilities, shelters, or detention facilities. Nineteen percent reported spending most nights in parks, streets, tents, cars or abandoned buildings. Fifteen percent of participants felt unsafe or very unsafe where they were staying\in the month preceding the survey. 

### 3.3. Substance Use

Data from the medical records yielded specific information on lifetime use of substances. Overall 17% were noted to have poly substance abuse in the past (e.g., cannabis, synthetic cannabis, cocaine, ecstasy), whereas 54% had used cannabis or synthetic cannabis only; and 6% had abused prescription drugs. Tobacco use was noted in 48% of cases and 8% of the residents reported ever binge drinking (5 or more drinks in one sitting). The HTST section on substance abuse only asked about use in the past month. Participants reported use of illicit drugs, including cannabis (40%), prescription or over-the-counter drugs (4.2%), and binge drinking (18%). 

### 3.4. Abuse and Sexual Exploitation

Childhood abuse data was obtained from medical records and a portion of HTST, which asked about abuse as a reason for running away from home. Thus, abuse data from the HTST is not available from participants who reported never running away from home. From the medical records, abuse was noted in 55 (42.6%) of the sample, of which a majority (*N* = 34, 61.8%) was physical abuse, followed by emotional/verbal abuse (*N* = 23l, 41.8%), and sexual abuse (*N* = 22, 40%) (See Table 2). Twenty-two (40%) of those with history of abuse had endured two or more types of abuse. The HTST data on the 54 participants with a history of running away from home revealed that abuse was listed as the reason for running away in 35 (64.8%) of the cases. Of these abuse cases, 25 (71%) were physical abuse, 28 (80%) were emotional/verbal abuse, and 5 (14%) were sexual abuse. Eighteen (51%) of participants who were abused reported more than one type of abuse on the HTST. 

The HTST included specific questions regarding trade sex. Based on this data, 27 (22.5%) of the youth reported ever trading sex for food, clothing, money, shelter, favors or other necessities for survival on their own. The average age of youth when they first traded sex was 17.4 ± 1.5 years, of which 19 participants (70%) were minors (age less than 18) the first time they traded sex. In the medical records review, trade sex was only documented in three (2.3%) cases with one documentation of the first age of trading sex as 19 years old (Table 2). Based on this question alone, 15.8% of the sample meets the definition of sex trafficking as trading sex under the age of 18. 

**Figure 1:**
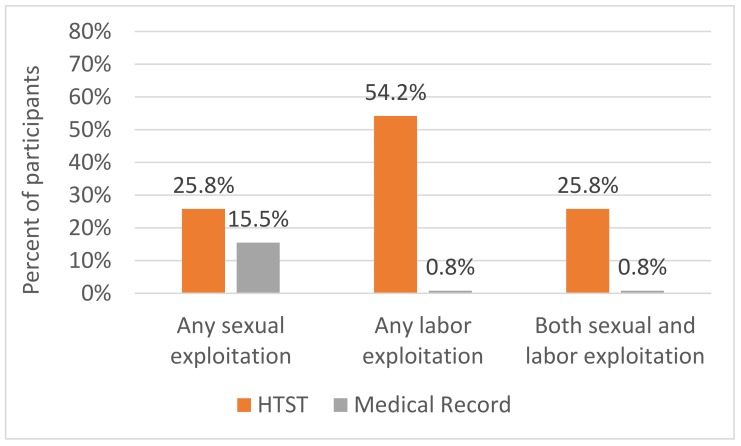
Identification of sexual and labor exploitation using the Human Trafficking Screening Tool (HTST) versus medical record.

When measuring forced sexual activity/commercial sexual exploitation, the HTST data were based on questions about acts of sex forced or encouraged by those considered to be employers or their associates, with a broad definition of work. These included the use of photos/videos on the internet, use of sex as business or personal favors, or forced trade sex in a variety of settings, including informal, escort services, brothels, massage establishments, or strip clubs. The medical records did not systematically include these queries. Therefore, data on forced sex was extracted based on various documentation in the medical record that could include sexual assault and in some cases childhood sexual abuse. Based on this data, commercial sexual exploitation was significantly more likely to be identified by the HTST; 31 participants (25.8%) indicated commercial sexual exploitation whereas the medical record documentation of forced sexual activity occurred in 20 (15.5%) of the sample (*p* = 0.04). A substantial proportion of participants (25.8%) reported experiencing both sexual and labor exploitation (see Figure 1).

### 3.5. Work-Related Exploitation

Table 3 contains details on labor trafficking divided into force, fraud, and coercion asked according to the HTST. No specific questions related to work conditions or employment practices were routinely recorded in the medical records. Thus work-related exploitation was significantly more likely to be reported using the HTST where 65 (54.1%) of participants reported some form of labor exploitation first experienced at an average age of 16.3 ± 3.0 years. In comparison, the medical record review showed significantly more documentation of labor exploitation than the HTST (*p* < 0.01). Of note, none of the foreign-born participants reported force, fraud, or coercion during the process of emigration to the United States. Thirty-one of those who reported labor exploitation reported ever trying to get help for the situation, most commonly from friends (20, 64.5%) and parents (14, 45.2%). In comparison, only eight youth (25.8%) reported asking for help from police or health care professionals. Twenty youth (64.5%) sought help from more than one source. 

### 3.6. Qualitative Findings

Five focus groups were conducted with 42 participants aged 18–21 years; 29 (69%) males and 13 (31%) females. The focus groups were designed to assess knowledge of youth regarding definitions of human trafficking, needs of survivors, perceived barriers to seeking services, and knowledge regarding available services. Findings from the thematic analysis are presented below. 

#### 3.6.1. Definitions

Participants provided 23 responses when asked about the definition of human trafficking. The most common component was the belief that human trafficking involved sexual exploitation through prostitution, either willingly—for survival—or unwillingly, through force or enslavement. As one participant commented *“So human trafficking is there’s a ho, and then there’s a pimp, and he’s pimping out the girl for some money”*. (FG2) Another participant stated *“I heard that human trafficking is basically prostitution”*. (FG2). A third participant differentiated human trafficking from prostitution, explaining *“I mean, human trafficking is generally—most definitely different than prostitution. Yes, they fall underneath—a close category but—human trafficking is when you’re forced to do—these acts not willing”*. (PG3)

Other definitions of human trafficking provided by participants focused on the idea of being forced, enslaved or sold. According to one participant, *“I’ve heard that it’s basically people who are getting kidnapped, particular women who are getting kidnapped, and sold to different people for sex, like slaves. It’s like a modern form of slavery”*. (FG5) As stated by another participant, *“Human trafficking is like rape and a bunch of different like drug dealing and like selling people to different various places and stuff”*. (FG1) Another explained *“The definition of human trafficking is—obtaining by means of force or—by trade another human being and selling that person for profit for yourself”*. (FG3)

#### 3.6.2. Vulnerability

Participants identified the need for survival and homelessness (12 and 11 quotations respectively) as the most significant factors in one’s vulnerability to becoming a victim of human trafficking. However, 23 quotations also described how family members were responsible for first engaging youth in human trafficking, particularly sexual exploitation.

Perpetrators were described as being acutely aware of when a youth was homeless or struggling to survive, as illustrated by this quote: *“The predators or the people who are looking for the prey—they see vulnerability. They can always tell when someone’s vulnerable”*. (FG 1) From personal experience, one participant reported *“He was really trying to like take advantage of me. Saying, I’ll give you $25 or something because I was like—I barely had something to eat the day I met that dude”*. (FG 1) Another participant acknowledged *“Sometimes you’re being forced to or you feel as though you have to, and that’s the only way you can obtain what you need out of life, either for yourself or for someone else”*. (FG 3) The idea of being taken care of in exchange for favors is exemplified in the following quote:

*“So it was this one time when I was homeless … I was walking down the street and this guy pulled over. He was like, ‘Get in my car.’ I was like okay because I’m thinking he fixing to give me ride to the police station. And I told him my story. And he was like, ‘You know, I’d take care of you.’ And then he pulled out his thing and was like, ‘If you do this for me.’ And then he was like, ‘I’ll buy you this food but then you got to do something else for me’”*.

(FG 2)

While many participants described scenarios in which they or someone they knew were approached by strangers, family members were also identified as getting youth involved in human trafficking activities. As one participant reported: *“I have … a friend whose mom used to—try to force her to stay with her uncle—to where—her uncle would do things to her. And then, she didn’t want to do that so, eventually, she moved out”*. (FG 3) Another described *“My father started pimping me up when I was sixteen. And I remember it being—absolute hell…. I honestly never…thought it was possible for a father to do such a thing to his own child”*. (FG 3) A third explained, *“My mom put me into it when I was younger but, at the time I didn’t know what it was but, I still knew it wasn’t right. But, yeah—what my mom really didn’t have a lot of stuff so to get money like to pay for bills and pay for stuff, like she would put me and some of her other children into it…”* (FG 4)

#### 3.6.3. Knowledge Sources

Participants identified school (e.g., health or physical education classes; 16 responses) and media (e.g., television, movies; 10 responses) as the primary sources of information when they first learned or heard about human trafficking, generally between the ages of 12 and 16 years. One participant reported *“I heard about human trafficking for the first time…in class…. And…they said young women were raped and then… sold for either drugs or money or housing or whatever”.* (FG1) Another explained *“I was watching TV and there was this thirteen-year-old girl, she ran away from home and…she started talking to people or whatever and she got kidnapped on her way to the other state. She got found a few months later and when they found her it exposed a major human trafficking ring that was going on around that area”.* (FG1) A third participant commented *“I first heard about human trafficking from the movie ‘Taken’”* (FG2), while a fourth explained *“I watch Law and Order. So there was this one episode where they was getting girls that run away from home, who pick them up and promise them all this stuff, and they’ll throw them in this abandoned place, and the girls will work for them, and they’ll get raped and stuff”*. (FG2)

#### 3.6.4. Experiences

Participants described situations in which they had experienced sexual (*N* = 3) or work (*N* = 3) exploitation, were approached to participate in a potentially exploitive arrangement (*N* = 8), or had family members involved in sexual exploitation (*N* = 4). One participant described being approached by a stranger: *“This dude…drove by me like for a couple, three times asking me questions. Do I mess with boys? Do I do anything to boys? Then he pretty much tried to get me in his car to go somewhere”*. (FG1) Others explained how families had either perpetrated or experienced sexual exploitation. For example, one participant reported *“My oldest brother, he used to be a pimp so I like always been around it. He always like brings females to the house and it just always been a factor in my life….”* (FG 4) Another commented: *“I heard about human trafficking from my sister because she was actually part of this*”. (FG 2) A third relayed, *“I heard about human trafficking when I was little because my mama got caught up in it when I was younger and now, my little sister has gotten caught up in stuff….”*. (FG 4)

Nine participants described someone they knew, outside of family members, who had been exploited—nearly all of these involved forced sexual activities. Three described witnessing some form of exploitation. One participant described *“I had a close friend who went missing one day. He was later found when I was in high school, and he—we found out that he had been a part of human trafficking. He was sold, and he was found in India”*. (FG 5) Another explained, *“I knew a victim of human trafficking in my freshman year of college. …there’s a common misconception that people get kidnapped forcefully, which is one of the ways. But, another way is people get lured away with the dreams of a better life and sometimes during sticky situations and under pressure, they yield, only to find out they are going to get trapped”*. (FG 5)

#### 3.6.5. Negative Outcome Expectancies

Participants identified fear and lack of trust as major barriers to seeking help, particularly police and healthcare provider mistrust, as well as stigma. A sense of violated trust leading to overall mistrust is captured by the following quotation: “*Well honestly if I had to trust somebody it would pretty much be me because I’m—really wouldn’t choose anybody else. Because like I’m pretty much the most trustworthy person I know. …I’ve tried in the past to trust so many people but at the same time I’ve been let down. So that made me not want to trust a lot of people….It’s just I’ve lost a lot but I’ve gained so little”.* (FG1)

Similarly, another participant explained: “Honestly, after being through such traumatic things it is very hard to trust anybody. …After having somebody so close to me—who was supposed to always protect you—do such a thing, it’s really hard to trust anybody. So, I’ve learned to be careful with who I trust”. (FG3)

#### 3.6.6. Police Discrimination and Mistrust

Twenty-two quotes described fear-related reasons for not seeking police help. Barriers cited by participants included fear of arrest, humiliation, or other maltreatment and lack of trust due to prior encounters with law enforcement or perceptions that certain police were “crooked” and might worsen the exploitive situation for their own gain. One participant explained it like this: *“…someone wouldn’t go to the police is because they might be scared or they just might think that the police might send them to jail for doing what they were forced to do or they just might not trust the police. Or they just think that the police are going to judge them and basically humiliate them while they’re out on the streets or not help them at all”*. (FG1) Another illustrated this point by referencing personal experience: *“… I’ve been a part of this group that three people was sexually harassed by one person. And we went to the police station to file a report and they didn’t do nothing to the dude. And he was actually at the police station that day and they let him go”*. (FG 2) A third spoke in more general terms about not trusting the police: *“There are some cops that is going to take advantage of the situation that you was in to get some more money because I guess they feel like they ain’t being paid enough or whatever. So you know—I don’t trust no cops period”*. (FG4)

Participants also described prior experiences with policemen that lead to general distrust. One participant explained, “So I kind of have trust issues so I—and I’ve been in situations where policemen have stopped me for no reason or—not to get on that whole black—I’m not feeling to get on all that. But I’ve been in situations numerous times—policemen have stopped me and harassed me physically. Thrown me into walls and stuff when I wasn’t doing anything. So it’s kind of just trust issues”. (FG 1)

Participants also described what they perceived as a dysfunctional legal system that moved too slowly to effectively prosecute perpetrators. As one participant explained, “*Another reason why I wouldn’t use the police is because, not only is the wait time long and outrageous, they want you to have so much documentation before they would even get a case worker or get an officer on your case. And it’s time consuming to get all that documentation. And you have to work around other people’s time when they can do it or whatever the case may be. And in most cases, they will be like, “Oh, we can’t help you because the time has lapsed”*. (FG 2)

#### 3.6.7. Healthcare Provider Mistrust

Feeling uncomfortable with psychiatric evaluation and associating mental health providers with other authority figures was discussed in ten quotes as a barrier to seeking help. Participants also disclosed mistrust in mental health providers’ genuine care and concern for them, and were hesitant to take prescription medications. As one participant explained, *“The only reason I don’t like psychiatrists is it’s their job. When I hear job, I just don’t like that word. Something about that just makes it seem like you don’t really care about me, personally. You care about the money that’s coming“.* (FG2) Another participant commented, *“I would say doctors should not see their patients as moneymakers but actually try to get to know them on a case-by-case basis. Take some time, don’t rush them. Make them feel comfortable. Build trust”*. (FG 5) A third participants offered *“One thing that would definitely get me to share and be more friendly towards the medical professional is if they were to share something that happened to them”*. (FG 5)

#### 3.6.8. Stigma

Perceived stigma associated with being homeless or engaging in survival sex was cited as a barrier to seeking help in 9 quotations. As one participant explained, *“They might not want to tell nobody because they are scared that people might look at them differently or they might just not want to talk about their experience. They just might want to forget about it all together because it’s pretty bad”.* (FG 4) Another participant commented, *“Some people are embarrassed about saying that they in a program about being homeless. Some people don’t want to tell nobody because it’s just—like they’re scared to tell people”*. (FG1) A third participant noted trust issues: *“I don’t think people will go (to the police) because they don’t want nobody to know they business or they might be ashamed or embarrassed. Even though it ain’t they fault. You don’t want other people to know what happened to you because you don’t know if they going to tell somebody or you don’t know if they going to try to do something”*. (FG 2) In order to overcome stigma associated with being homeless or engaging in survival sex, one participant suggested *“You have to like know your self-worth and know that you’re not as—I don’t know how to put in words but, you’re not as bad as everyone makes you seem as the person that’s keeping you and is making you seem”*. (FG 5)

#### 3.6.9. Perceived Resources and Needs

When discussing what participants knew about available local resources for victims of human trafficking, local shelters were discussed in 11 quotations, followed by family and friends (6) and places of worship (3). In contrast, medical clinics and the police were only mentioned in one quotation each. As illustrated by the following quotations, specific services participants’ perceived that victims needed included jobs, shelter, food, safety, and proper identification: *“Maybe like a job and a place to sleep…where you have a shower, food…Knowing that you don’t really have to worry about people coming in and messing around with your body or messing around with your life…”* (FG1) *“I think they might need counseling, job preparedness or job readiness, and also, housing”.* (FG 2) *“…a safe place for them to go without any serious questions being asked…. Just a place for them to go—be like—‘This is my situation, I need this help’—and—go from there”*. (FG 3) *“…that’s the most immediate thing you can do—get them out of that environment and look for a place for them to stay”.* (FG 5)

Participants also recognized the need for long-term and intensive mental health services for rehabilitation (nine quotes). As one participant explained, “…some people like myself included, end up getting PTSD afterwards. So they end up needing to see like therapists and stuff and get on meds and different stuff like that and some people go through worse things so they kind of be scarred for life so they have to stay in therapy and stay on meds and different stuff for a while”. (FG 4)

## 4. Discussion

The inconsistencies in screening measure sensitivity for human trafficking among youth experiencing homelessness found in this study is notable and represents both research and healthcare practice gaps that warrant attention. This study seeks to address these gaps in the literature by comparing the effectiveness of a standard youth assessment tool verses a specific screening tool for human trafficking by healthcare providers among a sample of high-risk youth seeking shelter and homeless services in Houston, TX. Providers at shelters are an underutilized yet strategic partner for identifying youth affected by human trafficking. Identification is the first step to engaging youth in secondary prevention and treatment for the sequela of human trafficking.

The findings from this study expand on the emerging literature regarding screening for human trafficking. The HTST was significantly more likely to identify sexual exploitation and labor exploitation than a commonly used psychosocial assessment tool (HEEADSSS). The HTST was also more sensitive to identifying risk factors for experiencing human trafficking, including youth involvement in the foster care and justice systems and running away from home [2,3,4,5]. HTST participants reported initial exposure to exploitive labor practices at younger ages than those exposed to sexual exploitation. Additional work is needed to explore factors that push young adolescents into the work force, including informal employment settings with lack of regulation which could put them at greater risk for experiencing labor exploitation. Youth commonly reported experiences of withholding all or some of promised pay. Nearly, 20% of youth also reported physical harm, restraint, and movement and communication restrictions which imposes further trauma and degrades trust. 

In contrast to prior studies on the prevalence of human trafficking among youth presenting to 10 shelters for services (19%) [6], we found substantially higher reports of both sexual and labor exploitation using HTST, with 25% of youth experiencing sexual exploitation and 54% experiencing labor exploitation [6]. These findings suggest that HTST is a substantial improvement over a commonly used assessment in determining past and current sexual and labor exploitation among youth seeking shelter or services at a homeless youth shelter-based healthcare clinic. A structured screening tool with questions specifically geared to identifying exploitation and risk factors is more effective at detecting both than a more general psychosocial screening tool. One strength of the HTST is that it asked about specific situations that allow a participant to disclose exploitation even if they did not have previous knowledge of trafficking definitions. This is a critical finding given the high prevalence of human trafficking experienced by youth served by homeless youth service and healthcare providers. Since healthcare providers of adolescents and young adults are frequently using HEEADSSS to conduct their consultation, many missed opportunities to identify and connect youth to prevention and treatment services likely exist [17]. While there is value in using an interview style with open ended questions, such as HEEADSSS, this data suggests that incorporating specific questions directed at human trafficking into the HEEADSSS assessment may minimize missed opportunities to identify and connect trafficked youth to prevention and treatment service. Therefore, in high-risk populations, using a structured human trafficking screening tool in addition to HEEADSSS may improve detection and subsequent treatment for exploitation. Additionally HEEADSSS does not inquire about labor exploitation, harassment, and safety despite the high-risk nature of youth homelessness [6]. Use of more sensitive and effective assessment tools, such as HTST.11 could help maximize the likelihood of connecting vulnerable youth with needed human trafficking-related preventive and treatment services. Of note, HEEADSSS was more effective than HTST at screening for substance use and mental health concerns. Therefore, this data suggests that the HTST coupled with HEEADSSS is recommended. Another notable finding was the percentage of participants experiencing more than one form of exploitation. Increasing awareness among providers about the need to screen for both sexual and labor exploitation is crucial, particularly when youth have experienced either.

Finally, the qualitative data provides further insight into the barriers to disclosure of experiences of human trafficking. Participants reporting histories of trauma suggested that this contributed to their sense of mistrust of other individuals and institutions, which acted as a barrier to seek help when needed. Being exposed to exploitation from early adolescence at a critical stage of social and emotional development can have lasting effects in their perception of safety and ability to access resources. Specifically, we found that youth often withhold reports of sexual and labor exploitation out of mistrust of the system, fear of involving the police if reported, not wanting to interact with the mental healthcare system, and stigma over the circumstances surrounding their involvement in human trafficking. This is also found in the triangulation with the HTST data that youth report low disclosure of human trafficking to the police and healthcare providers. Many states treat commercially sexually exploited adolescents and young adolescents as criminals. This means that the ramifications of disclosing their exploitation may include arrest, detention, adjudication, conviction, incarceration, and a criminal record [22]. Providers can address these barriers to disclosure by openly discussing resources available to youth who have been exposed to human trafficking and describing the process and procedures of accessing resources using youth friendly, non-judgmental, and inclusive communication strategies. Mistrust of law enforcement can also be addressed by incorporating medical legal partnerships into clinical settings serving youth experiencing homelessness. These legal advisers could act as advocates for potential survivors of trafficking and provide the support needed to navigate disclosure and reporting systems and to assist in accessing needed resources for safety, housing, and treatment.

## 5. Limitations

While the findings from this study are a critical next step to improving the identification of and treatment for human trafficking among youth experiencing homelessness, there are several limitations that should be considered when interpreting the results. Youth in this study were recruited from a single shelter in Houston Texas. Results from this study may not generalize to other shelters serving youth or regions of the country. In addition, the sample reflects youth who are connected to sheltering and healthcare services. Youth who experience homelessness who do not access services from a shelter may be at increased risk for sexual and labor exploitation and warrant further research.

## 6. Conclusions

Healthcare providers who work with adolescents and young adults experiencing homelessness are critically important to identifying youth experiencing human trafficking, yet they often lack specialized training in recognizing human trafficking and addressing the needs of these youth [4,7,23]. Evidence continues to suggest that youth experiencing human trafficking interact with the healthcare and social services systems where they can be, but are often not, identified [1,8]. Further research is needed to determine best practices in training healthcare providers to assist youth in the disclosure of human trafficking and the explore best practices in post-disclosure experiences to address barriers to disclosure. Additionally, individual and family level interventions that provide education regarding various forms of exploitation, increase access to community resources to prevent the need for child labor, and increase social capital and trusted network of adults may help youth avoid trafficking and disclose trafficking to trusted adults.

## Figures and Tables

**Table 1:** Sample characteristics reported on the Human Trafficking Screening Tool (HTST) versus medical record review.

Characteristic	HTST (*N* = 120)	Medical Record (*N* = 129)	*p*-Value
Age, Mean ± SD	19 ± 1.0	19 ± 0.9	NS
	*N* (%)	*N* (%)	
Race ^ψ^			NS
Asian	1 (0.8)	0	
American Indian/Alaska Native	2 (1. 7)	0	
Black/African American	74 (62. 7)	97 (77.0)	
White/Caucasian	19 (16.1)	19 (15.0)	
Mixed Race	19 (16.1)	0	
Other	3 (2.5)	0	
Ethnicity			
Hispanic	17 (14.4)	10 (7.9)	
Sex/Gender			NS
Female	56 (46.7)	55 (42.6)	
Male	61 (50.8)	74 (57.4)	
Transgender	1 (0.8)	0	
Other	2 (1.6) *	1 (0.01) **	
Sexual Orientation			NS
Heterosexual	92 (76.7) ^≠^	59 (45.7)	
Homosexual	11 (9.2)	8 (6.2)	
Bisexual	12 (10.1)	8 (6.2)	
Other	4 (3.4) ^	0	
Not recorded		54 (41.9%)	
Highest education,			
12th grade or above	62 (51.7)	66 (51.2)	NS
Pregnant or parenting	31 (25.8)	24 (18.6)	NS
Ever arrested	53 (44.2)	17 (13.2)	<0.01
History of foster care	43 (35.8)	29 (22.5)	0.02
Age at first placement, Mean ± SD	8.3 ± 5.6	7.6 ± 5.2	NS
Kicked out of house by family	86 (71.7)	63 (48.8)	<0.01
Age at first episode, Mean ± SD	16.1 ± 2.3	18.3 ± 1.3	<0.01
Runaway	54 (45)	10 (7.8)	<0.01
Age at first episode, Mean ± SD	14.2 ± 2.6	NA	

* androgynous, gender fluid; ** male and female, ^ demi-sexual, not recorded; ^ψ^ Data on Race or Ethnicity not available on 2 surveys and 1 medical chart; ^≠^ Data on sexual orientation was not recorded on 1 survey; NA = not asked.

**Table 2:** Details of sexual exploitation * identified using the Human Trafficking Screening Tool (HTST) versus medical record review.

Assessment Tool Used	HTST (*N* = 120) *N* (%)	Medical Record (*N* = 129) *N* (%)	*p*-Value
Traded sex Ever traded sex Traded sex as a minor	27 (22.5) 19 (15.8)	3 (2.3) NA	<0.01
Forced sexual activity Has anyone you have worked for or their associates: Forced you to do something sexually that you didn’t feel comfortable doing Put your photo on the Internet to find clients for sex Forced you to engage in sexual acts with family, friends, or business associates for money or favors Encouraged or pressured you to do sexual acts or have sex, including photos or videos Forced you to trade sex for money, shelter, food or anything else through online websites, escort services, street prostitution, informal arrangements, brothels, fake massage businesses or strip clubs	20 (16.7) 9 (7.5) 14 (11.7) 16 (13.3) 13 (10.8)	20 (15.5) **	

NA = not asked; * Only survival sex at <18 years is considered sexual exploitation; ** Forced sexual activity identified using HEADDSS includes all forced sexual activities, including child abuse and rape.

**Table 3:** Work-related exploitation identified using the Human Trafficking Screening Tool (*N* = 120).

Has Anyone You Have Worked for or Their Associates:	*N* (%)
Force	
Physically forced you to do something you didn’t feel comfortable doing	21 (17.5)
Locked you up, restrained you, or prevented you from leaving	19 (15.8)
Physically harmed you (beat, slap, hit, kick, punch, burn etc.…)	25 (20.8)
Fraud	
Tricked you into doing different work than was promised	22 (18.3)
Made you sign a document without understanding what it stated	6 (5.0)
Refused to pay you or paid less than they promised	33 (27.5)
Coercion	
Restricted or controlled where you went or who you talked to	27 (22.5)
Deprived you of sleep, food, water, or medical care	11 (9.2)
Not let you contact your family or friends, even when you weren’t working	11 (9.2)
Kept all or most of your money or pay	25 (20.8)
Kept your ID documents from you	14 (11.7)
Threatened to get you deported	1 (0.8)
Threatened to harm you or your family or pet	9 (8.5)
Physically harmed or threatened a co-worker or friend	4 (3.3)
